# Tuning the Photophysical Properties of Ru(II) Photosensitizers for PDT by Protonation and Metallation: A DFT Study

**DOI:** 10.1021/acs.jpca.3c00839

**Published:** 2023-04-11

**Authors:** Maciej Spiegel, Carlo Adamo

**Affiliations:** † Department of Pharmacognosy and Herbal Medicines, Wroclaw Medical University, Borowska 211A, 50-556 Wroclaw, Poland; ‡ Chimie ParisTech, PSL Research University, CNRS, Institute of Chemistry for Life and Health Sciences, F-75005 Paris, France; § Institut Universitaire de France, 103 Boulevard Saint Michel, F-75005 Paris, France

## Abstract

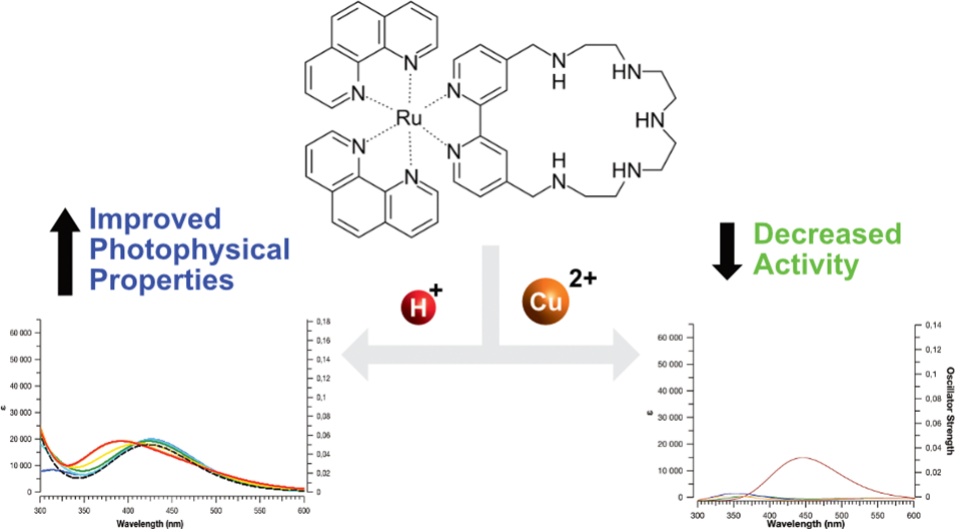

Due to their activity, photosensitizers with the Ru(II)–polypyridyl complex structure represent an intriguing class of photodynamic therapy agents used to treat neoplasms. However, their solubility is poor, intensifying experimental research into improving this property. One recently proposed solution is to attach a polyamine macrocycle ring. In this paper, the density functional theory (DFT) and time-dependent DFT (TD-DFT) studies on such derivative were performed to assess the impact of the protonation-capable macrocycle and its ability to chelate transition state metals, as illustrated by the Cu(II) ion, on the expected photophysical activity. These properties were determined by examining ultraviolet–visible (UV–vis) spectra, intersystem conversion, and type I and II photoreactions of all species possibly present in a tumor cell. For comparison purposes the structure devoid of the macrocycle was also examined. The results show that the subsequent protonation of amine groups improves the reactivity, with [H_2_L]^4+^/[H_3_L]^5+^ being borderline, whereas complexation appears to weaken the desired photoactivity.

## 1 Introduction

Compared to conventional approaches like chemotherapy or surgery, photodynamic therapy (PDT) distinguishes itself as a minimally invasive medical treatment and a viable alternative, having limited side effects, an easy procedure, and a short convalescence time.^1^ Intravenously administered photosensitizing agent (Ps) is preferentially absorbed and opportunely introduced in a specific tumor cell before being irradiated with low-energy tissue-penetrating light of the appropriate wavelength to activate the drug and induce its photodynamic effect. PDT is based on the light-induced, controlled generation of reactive oxygen species (ROS), the significant component of which is highly reactive singlet oxygen. ROS causes oxidative damage to biological structures of a cell and hence exhibits the potential to eliminate malfunctioning ones. This therapeutic strategy has been demonstrated to be effective in the treatment of different classes of cancer.^2^ It has also been found to help eradicate bacterial and viral infections^3−5^ and promote wound healing,^6^ and it has applicability for environmental purposes, such as water purification.^7^

A series of sequential photochemical reactions can explain the molecular mechanism behind PDT activity. The first is the excitation of Ps from its ground singlet state (S_0_) to one of the possible higher electronic states (S_n_) associated with the greater energy. Although the low-lying singlet state (S_1_) is rarely the brightest, most photoactive systems follow Kasha rules.^8^ Any higher excited state undergoes quick internal conversion (IC) to S_1_, which accounts for the apparent activity. The low-lying singlet state can then undergo a nonradioactive intersystem spin crossover (ISC) into one of the accessible triplet excited states (T_n_), linked often with a longer lifetime. The PDT agent activity strongly relies on the effective ISC that occurs due to spin–orbit coupling (SOC) between the two concerned states, ⟨S_n_|Ĥ|T_m_⟩, and so higher SOC values improve the kinetics of nonradiative transitions between states of varying multiplicity.^9^ Among other factors, the SOC values rise in the presence of atoms with large atomic numbers, a phenomenon known as the heavy atom effect (HAE).^10^

The newly populated triplet state can react directly with organic substrates producing intermediate radicals that form superoxides, peroxides, and hydroxyl radicals, upon interaction with molecular oxygen, capable of destroying cells. Such activity is referred to as a type I photoreaction. In any case, the other way, known as type II photoreaction, is more desirable. In its course, the triplet excited chromophore transfers its energy directly to the ground-state molecular oxygen, ^3^O_2_ (^3^∑_g_^–^), converting it to highly cytotoxic singlet oxygen, ^1^O_2_ (^1^Δ_g_). With a half-life of 40 ns in tissues and activity limited to a spherical region of no more than 10 nm diameter cantered at the point of origin, ^1^O_2_ behaves like a chemical scalpel, reacting quickly with surrounding biological targets, ultimately inducing cell death in a targeted manner—only in the tissues where the PS is accumulated.^11,12^ Worth underlining is that, depending on the electrical structure of the photosensitizer and the quantity of oxygen in cells, both processes might occur concurrently, which is typically troublesome in the hypoxic environment of neoplastic cells where PDT agents may be less successful. However, the fact that ROS do not rely on a single biological mechanism of action (unlike kinase inhibitors, for example) makes it more difficult for cancer tissue to develop resistance to them.

Some critical aspects of efficient Ps have been therefore formulated;^13^ namely, it must be: (i) stable, free of intermolecular aggregation, which would reduce its phototherapeutic efficiency, (ii) nontoxic in the dark, (iii) resistant to cellular redox reactions, and (iv) soluble under physiological conditions. The predominance of type II photoreaction over type I that has been demonstrated in clinical investigations to be associated with far more remarkable outcomes is also sought. Aside from the conditions mentioned above, an ideal PDT agent should also:^14^

- have an absorption band that falls within the so-called therapeutic window (500–900 nm) to ensure a deeper radiation penetration into tissues and, unlike UV-A, avoid side effects due to high-frequency vibrations;
- exhibit high ⟨S_n_|Ĥ|T_m_⟩ values to accommodate for a sufficient rate of ISC;
- possesses a substantial fluorescence triplet quantum yield (ΦT), resulting in significant ROS generation upon irradiation;
- have the energy difference between the ground state and lowest triplet excited state (Δ*E*_S_0_-T_1__) greater than 0.98 eV to generate ^1^Δ_g_ singlet oxygen in type II photoreaction.

With the surge of interest in photodynamic therapy, several chemical groups have been identified as possible photosensitizers. In particular, polypyridyl Ru(II) complexes were evidenced to possess reasonable physiochemical properties and improved photosensitizing effect, benefiting from HAE that facilitates high singlet oxygen quantum yield, long-lived emission dependent on the intracellular milieu, and photostability.^15^ Aside from these benefits, DNA intercalation and eventual exploit of the redox potential as a support for type I photoreactions have also been reported.

Nonetheless, these therapeutical agents are also burdened with severe limitations, including an absorption profile outside the therapeutic window, poor tumor selectivity, and negligible solubility. Some chemical modifications have been proposed to overcome those flaws.^13^

**Figure 1:**
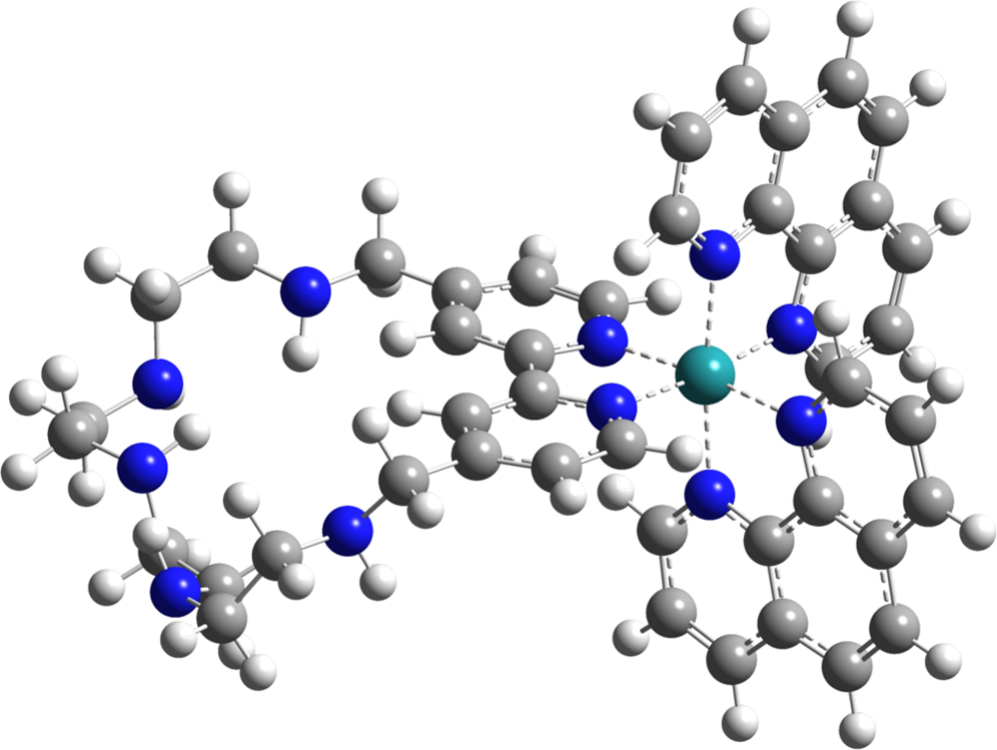
Optimized ground-state structure of L^2+^.

Very recently, Conti et al.^16^ synthesized and presented a new Ru(II) complex (L^2+^, Figure 1): it was evidenced through *in vitro* and *in vivo* studies to be highly soluble in water due to the presence of the polyamine ring, to possess reasonable cell–penetrating abilities, and to have photochemical characteristics essential for efficient ROS generation. Moreover, unlike previously suggested PDT agents, this one also revealed itself to be an excellent Cu^2+^ chelator. This feature is claimed to allow the macrocycle scaffold to assist in forming ROS *via* the metal-induced Fenton’s reaction. However such behavior would be in contrast to the established one for other systems with a similar fragment.^117−120^

The purpose of this article was to investigate the properties of such a Ru(II) complex using density functional theory (DFT) and its time-dependent variation (TD-DFT). We aimed to examine its photophysical properties and to shed new light on type I and type II photoreactions driving the observed activity. The presence of the macrocycle, the influence of protonation, and the eventual complexation of Cu^2+^ on the properties mentioned above were also discussed to grant the applicability of the theoretical conclusions.

## 2 Methods

All of the computations were done in the framework of density functional theory (DFT) and time-dependent density functional theory (TD-DFT) using default settings as available from the Gaussian16, rev. C.01 quantum chemistry software.^17^ Ground-state optimization was conducted under the regime of PBE0/6-31G(d) level of theory^18−20^ for all of the atoms except for ruthenium and copper, for which quasi-relativistic Stuttgart-Dresden effective core potentials and related basis set^21−23^ has been employed—the reliability of that choice in studies on metalorganics has been evidenced in other studies.^24−27^ Throughout the work, solvent effects were included employing integral equation formalism polarizable continuum model (IEFPCM),^28^ corresponding to a linear response in nonequilibrium solvation. Since the electronic absorption and fluorescence measurements have been obtained in an aqueous Tris–HCl buffer (5, 50 mM, pH = 7.2), water (ε = 2.27) has been considered a suitable solvent for the theoretical study to mimic the experiment. No symmetry constraints have been imposed during the geometry optimizations.

The photophysical properties and the feasibility of type I and type II photoreactions were examined with M06^29^ in 6-31+G(d)/SDD basis set, the feasibility of which was confirmed through the benchmark of 11 exchange-correlation functionals (APFD,^30^ B3LYP,^31−33^ B3P86,^32,34^ B3PW91,^34,35^ CAM-B3LYP,^36^ LC-ωHPBE,^37^ M06,^29^ M11,^38^ M062X,^29^ PBE0,^18^ and ωB97XD^39^). The chosen level of theory was evidenced to yield excitation energies the most comparable to the experimental absorption spectra. As a convention, λ_max_ corresponds to the absorption transition at the longest wavelength.

The water/n-octanol partition coefficients, log *P*, were estimated using free energies from corresponding solvents *via* a solvation model based on density (SMD) at 298.15 K. The standard free energy associated with the solute transfer from the aqueous phase (w) to octanol (o) was obtained using the equation:
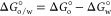
which yielded value then used to assess log *P* in the following manner:
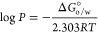
where *R* is a gas constant, and *T* is the temperature in K.

Spin–orbit matrix elements were evaluated with TD-DFT implemented in the Orca^40^ at their ground-state optimized geometries. For this purpose, M06 was coupled with the deMon2k-DZVP-GGA basis set.^41^ The spin–orbit couplings are defined according to the formula:
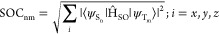
where Ĥ_SO_ is the spin–orbit Hamiltonian.

The amount of charge transfer (*q*_CT_) and associated hole–electron distance (*d*_CT_) have been calculated according to the methodology reported elsewhere^42^ to quantify the electronic redistribution induced by the excitations.

To compute the vertical ionization potentials and electron affinities of the lower-lying singlet (VIP, VEA) and triplet (VIP_T1_, VEA_T1_) states, the vertical energies of radical cations and anions have been obtained over the ground-state electronic configuration in the solvent.

The decision to use Kasha’s rule for internal conversion (KISC) instead of time-dependent vibrational coupling (TDVC) is primarily based on the complexity of the system being studied. Although TDVC provides a more accurate and comprehensive description of excited-state dynamics by considering the coupling between electronic and vibrational states, it involves solving the equations of motion for all of the particles in the system at every time step, which requires a significant amount of computational power in case of larger molecules.^43^ Moreover, the outcomes strongly depend on the thermal correlation function.^44^ In contrast, KISC is a more feasible option based on a simple energy threshold criterion.^8^ It does not require a detailed description of the vibrational modes or their coupling to the electronic states. While still providing a reasonable approximation of the excited-state dynamics, KISC is computationally efficient and, therefore, more convenient in the case studied here, as evidenced by its previous successful applications for investigations on Ru(II)-composed photodynamic therapy agents.^45,46^

## 3 Results and Discussion

### 3.1 Preliminary Concerns

Because of a polyamine ring, the structure of the investigated Ru(II) complex can be easily protonated in an aqueous solution when the pH decreases. These charged states may have different photosensitizing effects and metal chelation capabilities. While the authors offer experimental data on log *K*, they do not provide the routes of H^+^ affinities. The latter have been computed and reported in the Supporting Information (Table S1). While the experimental and theoretical findings show that [HL]^3+^ and [H_2_L]^4+^ are present at physiological pH in the grand majority (19.5 and 79.3%, respectively), one should keep in mind that the continuing inflammatory process within the cancer cell tends to reduce its pH; thus, the actual molar fraction may change, including the presence [H_3_L]^5+^ form in a non-negligible population. Therefore, a particular emphasis will be placed on explaining the future significance of the protonation state and thus considering the role of the polyamine ring, including the utterly deprotonated structure, hereafter denoted as L′ (Figure S1).

The next point to consider was selecting the most suitable computational protocol for TD-DFT investigations. To evaluate it, a set of exchange-correlation functionals listed in the Methods were examined in reproducing the absorption spectra and their principal bands. The best agreement was observed for the M06/6-31+G(d,p)/SDD level of theory (the details are accessible from Table S2), which was employed for all photochemical investigations.

### 3.2 Ground and Excited-State Structures

Geometrical properties of the optimized ground states (S), as well as the low-lying singlet and triplet ones (Table S3), demonstrate that the Ru(II) center is surrounded by ligands in a pseudo-octahedral symmetry regardless of the environmental pH. The N–Ru–N angles are close to 80°, while the lengths of Ru–N bonds are enclosed between 2.05 and 2.13 Å. Although only marginally, both properties diminish when H^+^ concentration increases in the milieu. The protonation of the macrocycle ring slightly affects Ru(II) close surroundings (Figure S2). However, the polyamine ring begins to fold upon protonation, which may impact the observed copper chelation capabilities due to the resulting steric hindrance. Finally, geometries of S_1_ and T_1_—the states prevalent in the PDT photoreactions—demonstrate no considerable structural divergence from those obtained for ground states.

**Figure 2:**
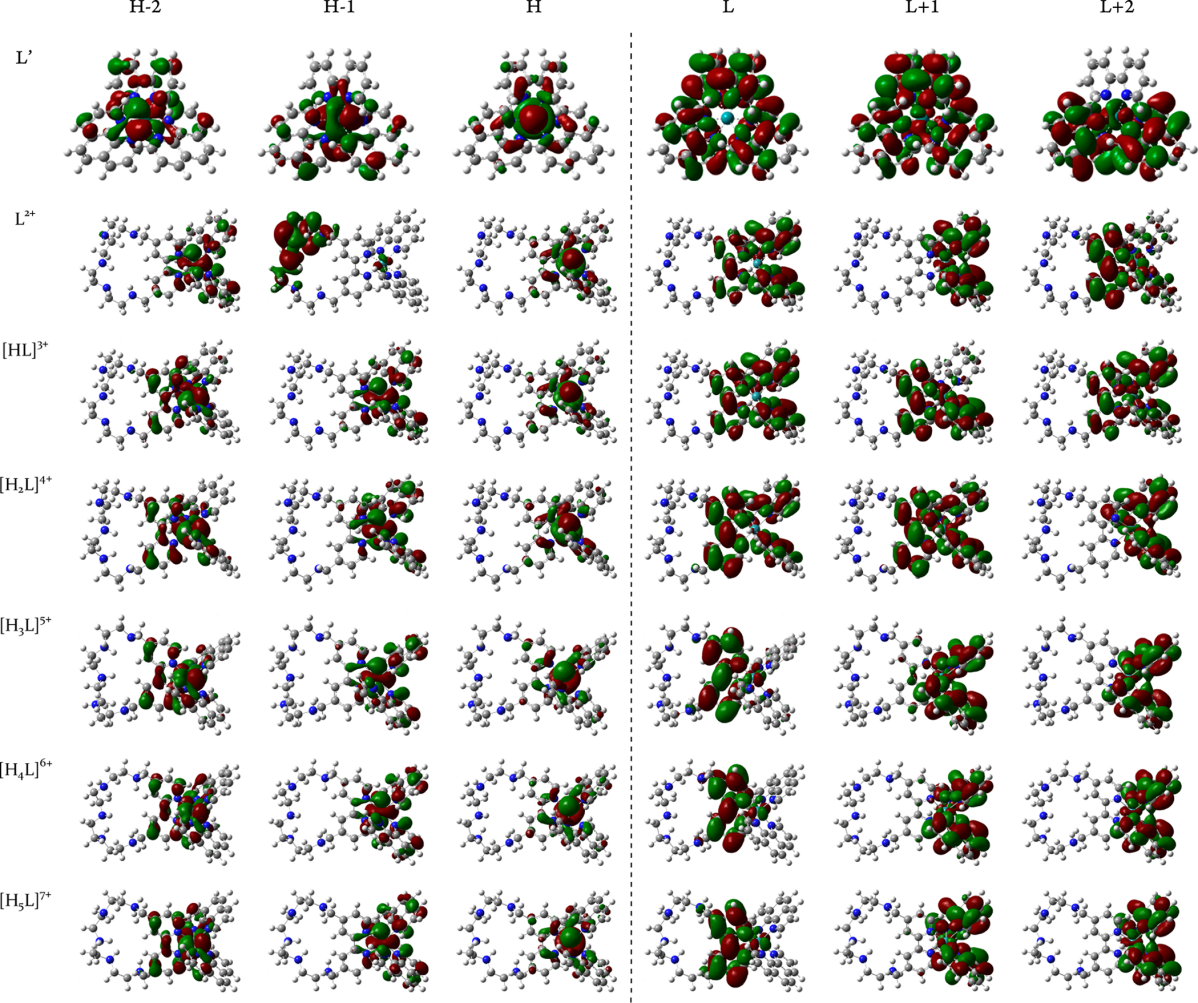
Graphical representation of the molecular orbitals involved in the transitions from the S_0_ categorized by the protonation state.

The electronic structures of the studied species’ ground states may be depicted by the mean of the most relevant frontier molecular orbitals, namely, HOMO – 2, HOMO – 1, HOMO, LUMO, LUMO + 1, HOMO + 2 (Figure 2). Table S4 displays the percentage contribution of the atomic orbitals stemming from Ru(II), 1,10-phenanthroline ligands, and 2,2′-bipyridilophane. The nature of the three mentioned occupied orbitals is mostly of ruthenium d-orbitals character, with HOMO exhibiting the largest one (83–84%), and this value remains constant across all species. Similar holds for HOMO-2, although with a lower contribution from the metal in exchange for the 2,2′-bipyridilophane. By sharp contrast, HOMO-1 changes noticeably, initially composed in 97% of 2,2′-bipyridilophane, a marginal 3% share of Ru(II) d-orbitals and a minor role 1,10-phenanthroline motifs in the case of L^2+^, to 3, 71–74, and 23–26%, respectively.

On the other hand, LUMO is built up of contributions deriving from 1,10-phenanthroline ligands in primary conditions but seems more vulnerable to proton attachment as it appears to benefit from increasing 2,2′-bipyridilophane contribution to the overall MO composition. Finally, it is worth underlining the notable variation in LUMOs composition discovered in L′. Although LUMO and LUMO + 1 of the other species are only weakly composed of Ru(II) atomic orbitals, they play a significant role in the case of this species. This shows that metal-centered (MC) charge transfer from t_2g_ to e_g_ orbitals is essential in the L′ excitation.

**Figure 3:**
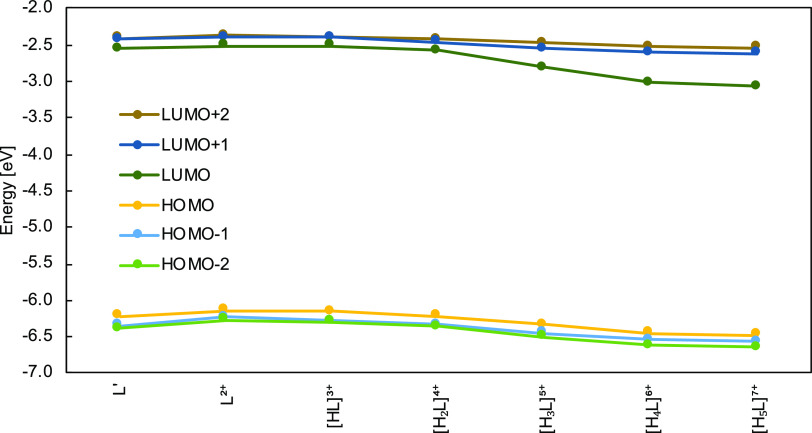
Calculated frontier orbital energies of ground states as a function of complex charge, in eV.

The influence of protonation is also seen in the energy trend plot depicted in Figure 3. A slight and sequential drop in MOs energies is apparent as a more charged state is considered. This is notably true for the LUMO, which experiences a considerable decrease in energy beginning from [H_3_L]^5+^ complex. Consequently, the HOMO–LUMO gap becomes smaller, and the excitations of these species are expected to require lower energy. Contrarily, LUMO + 1 and LUMO + 2 molecular orbitals are more resistive. Their energy difference between various protonated states appears smaller, which may be owing to the previously evidenced highly constant composition independent of the protonation state. As indicated by L′ results, the absence of a macrocycle does not appear to have significantly altered MOs energies compared to the original L^2+^ molecule, implying that mutual variations in photoactivity are minor.

To summarize the structural analyses, the Ru(II) binding energies in the coordination center were evaluated concerning the increasing charge of the system. The absolute and consecutive values are given in Table S5. The protonation process reduces the dipole nature of the investigated molecule and might destabilize its core component. The estimated data shows that the binding forces at the Ru(II) coordination center are affected by the pH, which lowering causes their drop by: 1.0 kcal/mol [HL]^3+^ < 3.8 kcal/mol [H_2_L]^4+^ < 9.7 kcal/mol [H_3_L]^5+^ < 13.6 kcal/mol [H_4_L]^6+^ < 14.2 kcal/mol [H_5_L]^7+^, with the binding energy for L^2+^ species designated as a reference point. Interestingly, in the instance of the L′ species, the Ru(II) appears to be stabilized by interactions stronger by approximately 3.5 kcal/mol than it happens for L^2+^.

One last item to confirm was the effect of protonation on solubility. Although a rudimentary estimate was used to assess the partition coefficients (log *P*), a reasonable trend was obtained (Table S6). The first point is that the L2+ system prefers the water milieu somewhat more than L′, but it is still unsatisfactory to consider it well soluble in polar solvents. However, as soon as the first amine group of the macrocycle accepts a proton, the solubility increases rapidly, and each subsequent protonation enhances the effect. Given that [HL]^3+^ and [H_2_L]^4+^ constitute the majority of the fraction present at pH = 7.4, the function of the polyamine ring becomes critical in the context of physiochemical properties, as already pointed out by the experiment.^16^

### 3.3 Photophysical Properties

In this case, incorporating organic chromophores, such as 1,10-phenanthroline and 2,2′-bipyridylophane, provides additional low-lying and long-living emissive intraligand triplet excited state (^3^IL) to the metal complexes. Furthermore, the presence of those motifs is connected with the occurrence of the metal-to-ligand charge transfer state (^3^MLCT), which stabilizes the Ps while decreasing the energy gap between the triplet and singlet states. As a result, the structure is more easily quenched by molecular oxygen and has a higher singlet oxygen quantum yield.^45^

**Figure 4:**
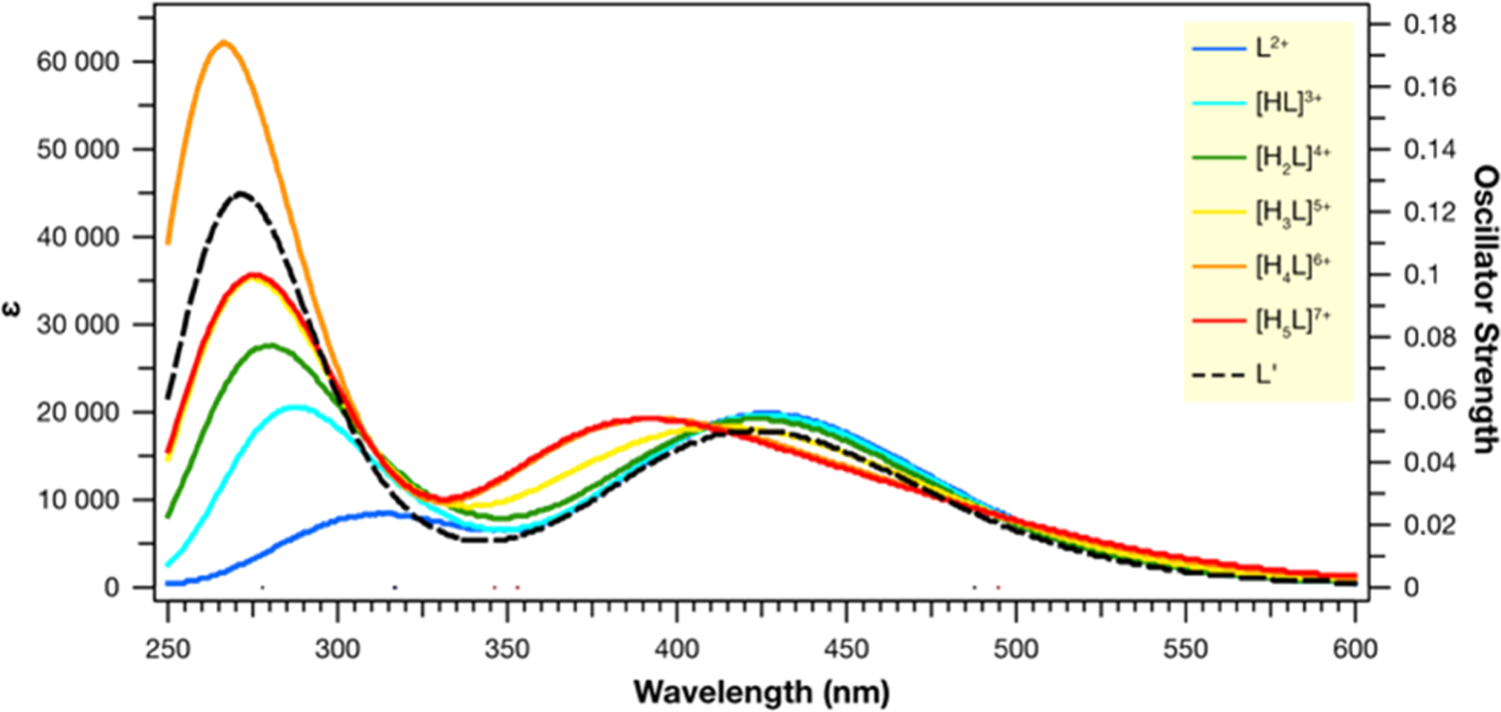
Computed UV–vis spectra of the studied species, in water, at the M06/6-31+G(d)/SDD level of theory.

To demonstrate quickly the effect of protonation degree and macrocycle presence on the absorption properties of Ru(II)-complex, the spectra corresponding to each charged state and compound devoid polyamine ring were modeled in water and are reported in Figure 4. Protonation, starting from [H_3_L]^5+^ species, causes a blueshift that well corroborates with the experimental findings. In contrast, the macrocycle itself does not affect the absorption properties. All of the structures exhibit two distinguishable bands: the most intense at ∼275 nm and another at ∼434 nm (or closer to ∼460 nm for the higher protonated forms).

The theoretically proven excitation energies for low-lying and brightest singlets (Table S7) were gathered, along with the corresponding transitions. The resulting singlet peaks correlate well with the experimental data (two intense bands are reported—at 450 and 380 nm), and the mean absolute error is less than ∼0.09 eV in the first case and less than ∼0.26 eV in the second. Two maximum deviations can be identified: for the first band, 0.13 eV, coupled with [H_5_L]^7+^, and for the second band, 0.23 eV, found for L^2+^. Even after applying a corrected linear response (cLR) approach for the solvent effects, these energies remained almost unaltered; contrary, external-iteration-derived wavelengths are significantly overestimated for the low-protonated species and, at the same time, notably underestimated for the highly protonated ones, yielding plausible results only for [HL]^3+^ and [H_2_L]^4+^ (see Table S8). All of these have been performed under the state-specific solvation calculation regime.

Transitions from H–3, H–2, H–1, H to L, L+1, L+2, and L+3 are primarily responsible for the brightest singlet excitations. The analysis of the MOs′ energies, with the degeneracy threshold of 0.001 Ha, has evidenced the presence of double-degenerated MOs: (LUMO + 1, LUMO + 2) in L′, L^2+^, and [HL]^3+^, as well as (HOMO – 3, HOMO – 4) in L′, (HOMO – 2, HOMO – 3) in L^2+^, and (HOMO – 1, HOMO – 2) in [HL]^3+^. This means that excited states produced by transitions involving those degenerate orbitals have also degenerated. All transitions can be characterized as MLCT excitations from Ru(II) dt^2^g or dz^2^ to either ligand. Several excitations, particularly those with higher energies, have a mixed character, also displaying IL from 2,2′-bipyridilophane to one of the 1,10-phenanthroline motifs. These ligand-localized transitions appear to be of ^1^ππ* charge transfer type.

In keeping with the initial findings on the MOs energy drop, particularly LUMO, during sequential protonation, lower-lying excited states become more accessible for higher protonated species and are associated with greater intensity. While the 450 and 380 nm bands are connected with excitation to S_9_ and S_12_, respectively, of L^2+^ and [HL]^3+^ forms, [H_2_L]^4+^ is based on a roughly equal contribution from the lower energy S_7_ (*f* = 0.125) and S_8_ (*f* = 0.113) states. The observed impact of increasing complex charge and the result of the “band split” in [H_2_L]^4+^ is evident for [H_3_L]^5+^ species, where the 450 nm band is coupled with single excitation even to lower-lying S_5_, while 380 nm stays characterized by S_8_. This is amplified for the latter two species, where S3 excitations cause the 450 nm peak.

### 3.4 Type II Photoreactivity

As mentioned, the radiationless intersystem crossing rate constant between S_n_ and T_m_ states is closely related to the generation of cytotoxic singlet oxygen. The spin–orbit coupling and the Franck–Condon weighted density of states (FCWD) modulate this process and are bound by the defined equation:
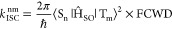
where the first term is proportional to the squared module of the spin–orbit matrix element between the associated S_n_ and T_m_ wave functions, whereas FWCD is proportional to the exponential factor in the context of Marcus–Levich–Jortner theory:^47^
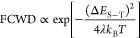
with λ being the Marcus reorganization energy and Δ*E*_S-T_ the energy gap between S_n_ and T_m_ states at their equilibrium geometries.

Estimating FCWD is, however, a computationally costly endeavor. Instead, the Fermi golden rule,^48,49^ which approximates ISC to the magnitude of the spin–orbit coupling values and the related Δ*E*_S-T_ energy gaps, is often used to forecast the deactivation routes that lead to the product of the ^1^Δ_g_ oxygen. It has recently been demonstrated^24^ that a rate for the nonradiative transition from the S_1_ to any lower-lying T_m_ state follows this. Furthermore, because internal conversion (10^–11^–10^–9^ s) and vibrational relaxation (10^–12^–10^–10^ s) are substantially quicker processes than intersystem crossing (10^–10^–10^–8^ s), the bulk of photoactive systems obeys Kasha principles underlining S_1_ → T_m_ ISCs as the major transitions of interest. Because the first bright state in all species analyzed is associated with relatively high energy, a quick decay to S_1_ is expected, and the related intersystem conversions are explored.

Table 1 displays the amplitudes of the SOCs and the energy gaps between the states under consideration, Δ*E*_S_1__-T_m_ and Δ*E*_S_0__-T_m_. Their corresponding transitions, alongside theoretical assignments, are shown in Table S9. The findings reveal that the kinetics of radiationless transitions vary significantly regarding the protonation state, as do the types of accessible paths. However, at least one observed SOC is substantial in all situations, reaching at least 300 cm^–1^. The energy difference value between the implicated excited states is never greater than 0.21 eV. The latter is especially advantageous for the ISC propensity when recalling the energy gap law, which is integrated into FWCD and states that the internal conversion rate between two electronic levels is inversely proportional to the amount of energy splitting between those levels. S_1_ → T_2_ of [H_3_L]^5+^ (506 cm^–1^), [H_4_L]^6+^ (522 cm^–1^), and [H_5_L]^7+^ (528 cm^–1^) are characterized by tremendous SOC values. At this point, it can be concluded that consecutive protonation, starting from [H_3_L]^5+^ species, reduces the number of available triplets to which ISC may lead.

**Table 1:** Spin–Orbit Matrix Elements (SOC, in cm^–1^) and Energy Gaps between Accessible Triplets^a^ and Low-Lying Singlet (Δ*E*_S_1_-T_m__ in eV) or Ground State (Δ*E*_S_0_-T_m__, in eV) for the Studies Species in a Water Solvent, Computed at the M06/6-31+G(d)/SDD Level of Theory

species	T_m_	SOC	Δ*E*_S_1_-T_m__	Δ*E*_S_0_-T_m__	species	T_m_	SOC	Δ*E*_S_1_-T_m__	
L′	T_1_	106.47	0.17	2.33					
	T_2_	129.88	0.17	2.33					
	T_3_	332.72	0.14	2.37					
	T_4_	64.98	0.10	2.40					
	T_5_	136.51	0.01	2.50					
L^2+^	T_1_	105.00	0.21	2.30	[H_3_L]^5+^	T_1_	124.17	0.15	2.18
	T_2_	155.40	0.19	2.32					
	T_3_	401.35	0.13	2.38					
	T_4_	51.46	0.11	2.39		T_2_	505.93	0.04	2.29
	T_5_	302.25	0.04	2.47					
[HL]^3+^	T_1_	105.78	0.21	2.31	[H_4_L]^6+^	T_1_	119.80	0.15	2.07
	T_2_	118.21	0.19	2.32					
	T_3_	47.65	0.13	2.38					
	T_4_	46.16	0.13	2.39		T_2_	522.46	0.05	2.17
	T_5_	311.79	0.04	2.47					
	T_6_	242.60	0.01	2.51					
[H_2_L]^4+^	T_1_	67.14	0.17	2.33	[H_5_L]^7+^	T_1_	105.49	0.15	2.04
	T_2_	121.68	0.16	2.34					
	T_3_	325.91	0.13	2.37		T_2_	527.54	0.04	2.15
	T_4_	48.08	0.09	2.40					
	T_5_	312.04	0.00	2.49					

a Five triplet states for L′, L^2+^, and [H^2^L]^4+^; six for [HL]^3+^; two for [H_3_L]^5+^, [H^4^L]^6+^, and [H_5_L]^7+^.

On the other hand, it notably enhances SOC, simultaneously decreasing the energy gaps, which shall account for their increased activity. The data also show that adding the polyamine macrocycle in going from L′ to L^2+^ species leads to a noticeable increase of the SOC values for the T_2_, T_3_, and T_5_ states, yet keeping almost the same energy differences. The net effect is an increase in the ISC mechanism.

The observed behavior can be rationalized using the El-Sayed rules,^50−52^ which foresee a substantial increase in SOC values when a symmetry change of the molecular orbitals involved accompanies the singlet-triplet transition. Furthermore, heavy elements, such as ruthenium, can boost their values via the HAE, as mentioned earlier, the impact of which grows as a function of the Z^4^ atomic number and is inversely proportional to the mean cubic radial distribution of electrons (r^–3^). Thereby, triplet excited states lying below S_1_ have been described to explain the noteworthy amplitudes of spin–orbit couplings—with relatively little error in reproducing experimental absorption data. edible Conceivable outcomes are also predicted to be yielded well.

According to the collected values, most triplet excited states engaged in the ISC are based on transitions from HOMO, HOMO – 1, or HOMO – 2 to LUMO, LUMO + 1, or LUMO + 2. As demonstrated (Figure S3), the electron density is transferred chiefly to more than one motif, primarily when higher triplet states are evaluated. Specifically, phenanthroline’s antibonding MOs are the top destination, though not always. Irrespectively, because the HOMOs engaged in such transitions are mostly Ru(II) d-type, and the LUMOs are π-type, the symmetry shifts, agreeing with El-Sayed principles and accounting for observable SOC values. A consistent pattern is seen when examining the change in the contribution from Ru(II) d-orbitals to one of the phenanthroline for a set of three consecutively protonated species—[H_3_L]^5+^, [H_4_L]^6+^, [H_5_L]^7+^. Despite the electron density sharing ultimate location on the phenanthroline motif for all of them, the transition from d_t^2^g_ (T_2_) has significantly higher SOC values than the transition from d_z^2^_ (T_1_). Similarly, and after careful consideration, it may be expected that spin–orbit coupling amplitudes are generally more significant when Ru(II) d_t^2^g_ is involved in the transition, as seen by their values for lower-protonated species.

It is also known that ISC is related to intramolecular charge transfer (ICT) if the latter takes mutually orthogonal electron-donating and -receiving components into account. The critical ICT process can stimulate the formation of efficient charge-separated states upon photo-excitation. While its presence and qualitative description have already been provided, it is sensible to supply the quantitative component to the effect (Table 2).

**Table 2:** Dipole Momenta Differences between the Ground and Excited States (Δμ, in *D*), Effective Distances (*D*_CT_, in Å), and Amount of Transferred Charge (*q*_CT_, in A.U.) of S_1_ and T_M_ States of the Studied Species Computed at the M06/6-31+G(d,p)/SDD Level of Theory in Water

species	state	Δμ	*D*_CT_	*q*_CT_
L′	S_1_	18.6308	2.717	0.871
	T_1_		1.427	0.628
	T_2_		1.180	0.676
	T_3_		0.603	0.669
	T_4_		0.322	0.661
	T_5_		1.171	0.683
L^2+^	S_1_	0.0626	1.698	0.840
	T_1_	–4.4019	1.422	0.644
	T_2_		0.980	0.640
	T_3_		0.947	0.661
	T_4_		2.367	0.722
	T_5_		1.233	0.691
[HL]^3+^	S_1_	0.4190	2.312	0.834
	T_1_	0.5731	1.409	0.637
	T_2_		0.734	0.635
	T_3_		1.608	0.696
	T_4_		0.288	0.668
	T_5_		1.222	0.689
	T_6_		1.640	0.703
[H_2_L]^4+^	S_1_	–0.4148	2.750	0.869
	T_1_	–0.4140	1.711	0.692
	T_2_		1.384	0.619
	T_3_		0.378	0.649
	T_4_		0.142	0.663
	T_5_		1.256	0.696
	T_6_		0.039	0.678
[H_3_L]^5+^	S_1_	–0.6584	2.819	0.885
	T_1_	–10.2855	2.692	0.795
	T_2_		3.065	0.885
[H_4_L]^6+^	S_1_	–2.0058	2.777	0.881
	T_1_	–11.0968	2.645	0.807
	T_2_		2.596	0.753
[H_5_L]^7+^	S_1_	–0.5114	2.786	0.882
	T_1_	–10.5177	2.659	0.810
	T_2_		2.606	0.756

The values of the dipole momentum difference between the excited state of interest and the ground state can be used as a rough evaluation of the ICT character of the transitions. Unless [H_4_L]^6+^ is considered, a small amount of charge transfer can be connected with S_1_ excitation (Δμ less than 1D) based on the calculations. In most situations, however, the difference in dipole momenta of T_1_ states is considerably more significant, reaching more than 10D in the case of the last three protonated states. Similarly, an immense value for L′ was obtained, which can be explained by the significant charge transfer. On the other hand, it is enclosed in a limited region, yielding the observed value. As a result, ICT looks to be the greatest for that compound.

The magnitude of the charge transfer within the molecule can be quantified more precisely by computing the DCT index, which corresponds to the distance between the average distance between the hole and the electron and the associated fraction of electrons transferred, denoted by *q*_CT_. For all S_1_, the amount of transferred charge and the accompanying effective distance confirm that ICT occurs. It does, in general, for most triplet excited states too. Still, for unique cases, such as T_3_, T_4_, and T_6_ of [H_2_L]^4+^, it is irrelevant due to the small distance pinpointing eventual local excitation nature.

The preceding results explain the prevalent SOC values for the computed transitions. The findings supplement those derived from El-Sayed rules, fully explaining the observed SOC values among the examined ISCs.

All the studied species have an energy gap between the ground state and the low-lying tripled excited state that is significantly greater than the energy required to excite molecular oxygen from its ^3^∑_g_^–^ to ^1^Δ_g_ state, indicating that the compound meets the essential criterion of an efficient PDT agent. The previously obtained experimentally recorded singlet oxygen quantum yield of 0.29 ± 0.06^16^ confirmed this, ensuring phosphorescence capacity.

Based on the data collected, a range of nonradiative decay paths are conceivable for L^2+^, and the most favorable routes can be distinguished as:
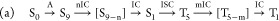

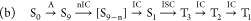
Similar ones involving S_7_ instead of S_9_ may be defined for [H_2_L]^4+^. Adequate pathways can be linked with [HL]^3+^; however, they are boosted by the presence of an extra T_6_ state below S_1_:
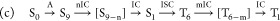
For protonated species, those paths are much more straightforward, with notably fewer internal conversions; the decay of [H_3_L]^5+^ can be explained by the given scheme:
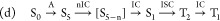
while for [H_4_L]^6+^ and [H_5_L]^7+^, it may go as:
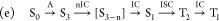
By evaluating the SOC values and energy gaps between the states involved in nonradiative intersystem spin-crossing, the optimal deactivation mechanism for each chemical may be hypothesized. The most conceivable decay of L^2+^, L′, and [H_2_L]^4+^ looks to be (b), but route (a) is also probably due to the minimal Δ*E*_S_1_-T_5__ value. Because SOC remains high and the energy difference between interacting states is mainly immaterial, [HL]^3+^ may use both (a) and (c) pathways. The last three species are fully expected to decay through ISC to T_2_, followed by IC to a low-lying triplet (routes (d) and (e), respectively).

### 3.5 Type II Photoreactivity

Another eventually relevant mechanisms of action Ps may employ are type I photoreactions. They entail the transfer of an electron or a hydrogen atom between the excited Ps (usually T_1_) and a substrate molecule, such as the cell membrane component or the sensitizer itself, to produce organic radical ions and free radicals. These radicals can then react with oxygen to generate oxygenated products such as O_2_^–•^ species, which can then participate in rapid bimolecular decay to yield other oxidizing agents—reactive oxygen species such as H_2_O_2_ and ^•^OH—capable of promoting reactions with biomolecules and amplifying cellular damage. The vertical electron affinity (VEA) and ionization potentials (VIP) of each molecule and molecular oxygen can be used to assess the feasibility of those processes.

Superoxide anion O_2–•_ can be formed by direct electron transfer: from Ps in its triplet state to ^3^O_2_ (1) or from the reduced form of Ps to molecular oxygen (2):
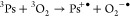
1
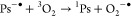
2The autoionization reactions that lead to the production of the Ps radical anion and radical cation via the reduction of its low-lying triplet state by the adjacent Ps itself in its S_0_ or T_1_ states can be summarized as:
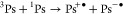
3
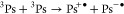
4The propensity of Reaction 3 can be determined by examining the computationally determined values of VEA and VIP of the Ps molecule in its triplet and singlet forms, the sum of which must be negative. Based on the estimated values, as shown in Table 3, Reaction 3 may undergo, and all of the species can be reduced by autoionization with neighboring Ps in the T_1_ state. This is because any absolute value close to the S_0_ VIP is lower than that of T_1_ VEA. The differences remain negative in all circumstances (−1.82, −1.84, −1.80, −1.64, −1.51, −1.48 eV for each subsequent protonation state). The VEA is significantly lower for the molecule without the polyamine cycle (−1.99 eV). At the same time, the triplet state of each investigated form can be reduced by the near T_1_ one via the autoionization Reaction 4, for the electron affinity of the triplet states are greater in 1.28, 1.30, 1.16, 1.03, 0.92, and 0.90 eV, in the similar order as previously. In this case, the L′ value is significantly higher (1.82 eV).

**Table 3:** Vertical Electron Affinities (VEA) and Ionization Potentials (VIP) Computed in Water at the M06/6-31+G(d,p)/SDD Level of Theory for ^3^O_2_^a^ and the Investigated Species in Their Low-Lying Singlet and Triplet Excited States

species	VEA(S_0_)	VIP(S_0_)	VEA(T_1_)	VIP(T_1_)
L′	–5.36	3.19	–5.18	3.36
L^2+^	–5.32	3.13	–4.95	3.67
[HL]^3+^	–5.36	3.15	–4.99	3.69
[H_2_L]^4+^	–5.40	3.24	–5.04	3.88
[H_3_L]^5+^	–5.50	3.53	–5.17	4.14
[H_4_L]^6+^	–5.60	3.74	–5.25	4.33
[H_5_L]^7+^	–5.63	3.79	–5.27	4.37

a VEA ^3^O_2_ = −3.67 eV.

To determine whether the Ps may follow the process (1), it should be verified that the sum of VEA(^3^O_2_) and VIP(^3^Ps) yields a negative value, and for the feasibility of process (2), the sum of VEA(^3^O_2_) and VEA (^1^Ps) must output the same sign. Given the proclivity of all species to receive an electron (negative VEA), the latter requirement is wholly met (−8.99, −9.03, −9.07, −9.17, −9.27, −9.30 eV in the protonation order and −9.0 eV in case of L′), implying that the reduced Ps, formed by photoreaction (4), can promote the electron transfer process to ^3^O_2_, generating the superoxide species. However, due to the larger ionization potential values of Ps in their triplet states compared to the electron affinity of molecular oxygen, the feasibility of O_2_^–•^ production by direct electron transfer from the ^3^Ps may be ruled out in all situations. As a result, the highly reactive O_2_^–•^ species can be produced by autoionizing Ps by photoreaction (4) and then transferring electrons to molecular oxygen in its ground state. The investigated species are also shown to become weaker electron donors as their protonation state increases, inhibiting direct electron transfer.

### 3.6 Copper Chelating Properties and Photochemical Properties of the Complexes

Copper, like iron, is an important element in the Fenton reaction producing free radicals. Its presence in the neoplasm cell—either natural or delivered by the molecule capable of chelating it—may be vital. Due to the parallel photoactivity of the PDT agent and H_2_O_2_ reduction by the given metal ions, the amplified generation of reactive oxygen species is a possible synergistic effect between those two. Moreover, the photosensitizer in its triplet or reduced form can regenerate, i.e., Cu^+^ ions through type I photoreactivity, and let them participate in the redox cycle once more. VEA of Cu^2+^ has been established to −1.23 eV. With that outcome, the reactions ^3^Ps + Cu^2+^ → Ps^+•^ + Cu^+^ is not feasible, whereas Ps^–•^ + Cu^2+^ → ^1^Ps + Cu^+^ represent a viable route.

Some basic photochemical properties of ground-state structures were studied to establish the performance of Cu(II)-PDT complexes. This is of particular interest also due to the presence of an unpaired electron on the 3d^9^ orbital and hence the paramagnetic nature of the ion, which in the experimental paper^16^ has been evidenced to account for the photochemistry of the ruthenium(II) polypyridyl structure.

At the outset, the Ru(II)-center geometrical parameters appear not to differ significantly from those established for the photosensitizer alone (Table S10). However, starting from [H_2_CuL]^6+^ species, a copper ion can no longer occupy the farthermost area of the macrocycle for the amino groups present there (NH-88 and NH-86, according to the numbering from Table S1) because they are protonated—the most plausible conformation becomes now is the one where Cu^2+^ is partially coordinated, just by two other groups, NH-70 and NH-72, which are in proximity to 2,2′-bipyridilophane scaffolds. It appears to have the consequences in slight and irrelevant changes on the Ru(II) center but a much more notable bent of the mutually planar aromatic pyridine rings. Due to the steric effects and distortion of sp geometry, photophysical properties might alter, as emphasized earlier in the text, due to their molecular orbitals’ contribution to the system’s absorption/emission properties. They are expected to be altered in the case of [H_2_CuL]^6+^. On the other hand, subsequent protonation leads NH-70 not to be involved in coordination and relaxation of the configuration, with Cu^2+^ being now found between NH-72 and NH-100.

The complex structure also has an entirely different electronic structure than the photosensitizer. Frontier molecular orbitals’ absolute energies and gaps are depicted in Figure S4. The energies of SOMO orbitals are the same in pairs [CuL]^4+^ – [HCuL]^5+^ and [H_2_CuL]^6+^ – [H_3_CuL]^7+^. While in the case of the latter pair, SOMOs of both species are localized at 1,10-phenanthroline motifs, in the former, one can find them on the ruthenium center ([CuL]^4+^) or copper center ([HCuL]^5+^). Similarly, complexation of Cu^2+^ and protonation yield entirely different localization of the discrepancies between those two pairs might be shown by examining β-spin LUMO orbitals which in all cases but [CuL]^4+^ are found on photosensitizer’s Ru(II) center. In the mentioned one, it is localized on copper chelation. Due to the differing MOs arrangement, although similar in both LUMO energies and gaps to the free photosensitizer species, different absorption profiles and photophysical properties are expected to be found among them.

**Figure 5:**
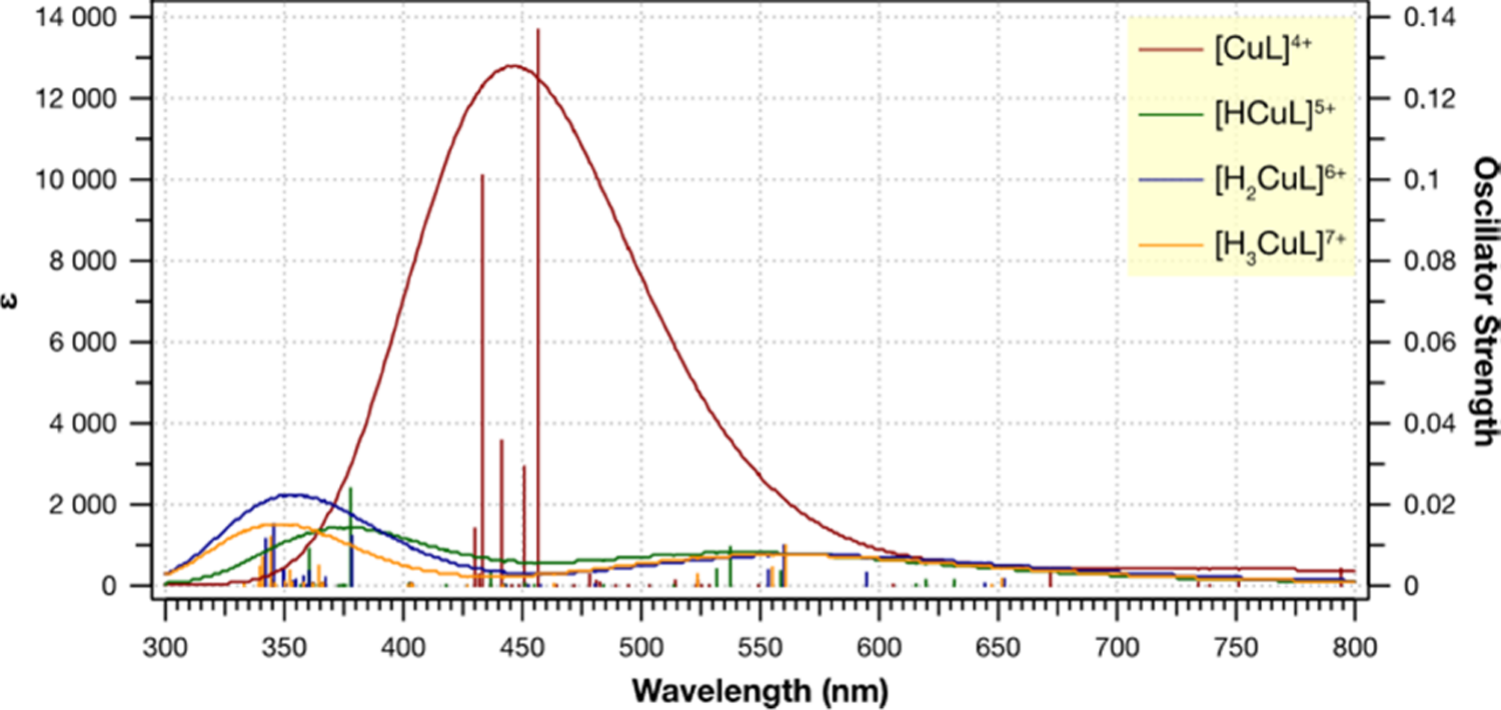
Superimposed UV–vis spectra of Cu^2+^-complexes at the different protonation states.

The theoretical absorption spectra have been computed to indulge further into the impact of copper chelation on basic photophysical properties (Figure 5). The first thing to note is a broad band found for [CuL]^4+^ and enclosed around 450 nm, which on the other hand, does not appear in the case of the remaining species. Comparing this outcome with the previously given spectra of free photosensitizer, the absorptivity dropped, and while the L^2+^ peak was found at around 20,000 units, here it is a little over 10,000. This underlines how the absorption profile of the remaining species has drastically dropped, for their values of ε are even smaller. Still refereeing to the previous spectra, two bands have been distinguished: at around 400–450 nm and approximately 250–300 nm. In this situation, a similar holds, at least for all but [CuL]^4+^; however, they become flattened and slightly red-shifted. After all, it appears that the chelation of copper ions alters the absorption profile and renders species less susceptible to electron transitions.

## 4 Conclusions

The photochemistry of the proposed and experimentally verified novel Ru(II) photosensitizer has been explained at the molecular level with theoretical chemistry methods. Density functional theory and its time-dependent version evidenced that protonation decreases the HOMO–LUMO gap and makes low-lying excited states more accessible. On the other hand, more ionized species have their Ru(II) destabilized to a greater extent. The presence of the polyamine macrocycle ring and its capability to change ionic state regardless of pH is essential. It skyrockets the solubility, a vital feature concerning the relatively poor bioavailability of those compounds. The best propensity in type II and type I photoreactivity has been found for the most protonated species. Due to the presence of the macrocycle, the compound exhibit also copper chelating properties. These have been found to have notably lowered absorptivity profiles, which might be correlated with their worse photoactivity.

## Supporting Information Available

Distributions of all possible ionic states; results of the TD-DFT benchmark; main geometrical features of the studied species; Ru(II) binding energies; orbital contribution to MOs; partition coefficient estimation; description of main transitions; comparison between experimental and theoretical data; and visualization of the structures and MOs involved in photoreactivity (PDF)
