# Supplementary material for: Tuning the Photophysical Properties of Ru(II) Photosensitizers for PDT by Protonation and Metallation: A DFT Study
Source: J Phys Chem A. 2023 Apr 11;127(16):3625–35. doi: 10.1021/acs.jpca.3c00839 (PMC10150361; doi:10.1021/acs.jpca.3c00839)
Supplement: Supplementary file 1 — jp3c00839_si_001.pdf [file jp3c00839_si_001.pdf]

Tuning Photophysical Properties of Ru(II) Photosensitizers for PDT by Protonation and Metallation: a DFT Study

Maciej Spiegel*,†,‡ and Carlo Adamo‡,§

†Department of Pharmacognosy and Herbal Medicines, Wroclaw Medical University, Borowska 211A, 50-556 Wroclaw, Poland

‡Chimie ParisTech, PSL Research University, CNRS, Institute of Chemistry for Life and Health Sciences, F-75005 Paris, France

§Institut Universitaire de France, 103 Boulevard Saint Michel, F-75005 Paris, France

Correspondance e-mail: maciej.spiegel@student.umed.wroc.pl

TABLES

Table S1. The distribution diagram computed using experimental log*K* values along with the protonation pathways of the L^{2+} macrocycle, the corresponding Gibbs free energies (in kcal mol^{-1}) and molar fractions (^{M}f) at pH=7.4, computed at the M06/6-31+G(d)/SDD level of theory.

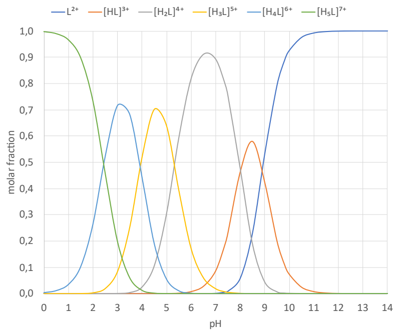

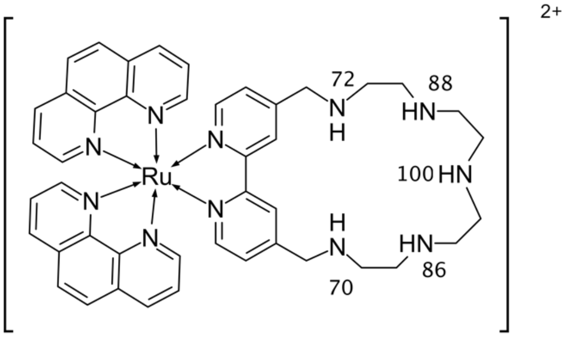

	L^{2+}	→	$[HL]^{3+}$	→	$[H_2L]^{4+}$	→	$[H_3L]^{5+}$	→	$[H_4L]^{6+}$	→	$[H_5L]^{7+}$
Site		NH-86		NH-88		NH-70		NH-72		NH-100	
ΔG°		-44.9		-35.7		-17.9		-19.4		-2.8	
logK		8.90		8.01		5.32		3.93		2.44	
^{M}f	0.62%		19.46%		79.27%		0.66%		0.00%		0.00%

To select the most appropriate functional to accurately describe the photophysical properties of the studied systems, a series of preliminary computations testing several XC density functionals against the reported experimental data were performed. The absorption peaks for L species in water calculated with M06 are in good agreement with the observed bands in the reproduction of the experimental absorption spectra (especially at high wavelengths), as can be seen in **Table S2**. The excellent performance of M06 has also appeared in earlier studies on metallic compounds.

Table S2. Comparison of theoretically estimated vertical absorption energies (λ^{A}_{theor}) with the highest oscillator strengths (f_{theor}) for L^{2+} excitations and experimental ones (λ^{A}_{exp}) for the two main bands, in nm.

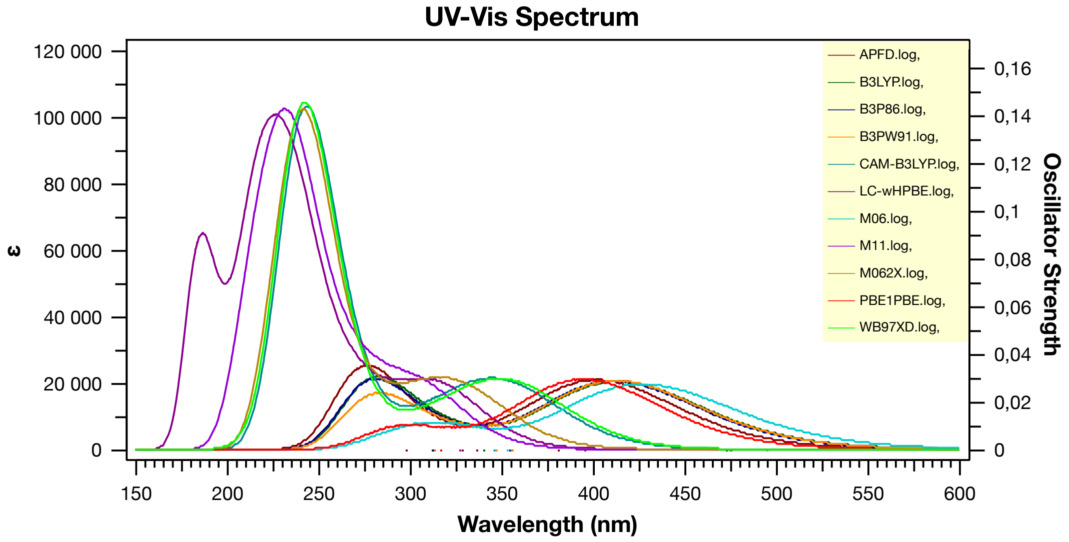

	APFD	B3LYP	B3P86	B3PW91	CAM-B3LYP	LC-ωHPBE	M06	M062X	M11	PBE1PBE	ωB97XD
$\lambda^{A}_{exp\_1}$						~450nm					
$\lambda^{A}_{theor\_1}$	407.44	419.86	417.52	418.39	352.02	310.15	433.93	331.33	306.02	399.62	355.79
f_{theor_1}	0.1801	0.1694	0.1766	0.1762	0.2125	0.1369	0.1598	0.162	0.1961	0.1644	0.2059
$\lambda^{A}_{exp\_2}$						~ 380 nm					
$\lambda^{A}_{theor\_2}$	—	—	—	—	317.66	—	323.29	—	—	322.44	319.35
f_{theor_2}	—	—	—	—	0.0736	—	0.0436	—	—	0.0275	0.0648

Table S3. Main bond lengths (in Å) and angles (in degrees) for the ground, S_1 and T_1 states computed at the PBE1PBE/6-31G(d) level of theory in an aqueous solvent.

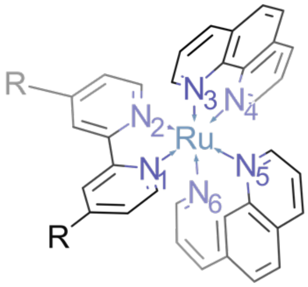

		Ru-N$_1$	Ru-N$_2$	Ru-N$_3$	Ru-N$_4$	Ru-N$_5$	Ru-N$_6$	N$_1$-Ru-N$_2$	N$_3$-Ru-N$_4$	N$_5$-Ru-N$_6$
L^{2+}	GS	2.06811	2.06695	2.07629	2.08170	2.08143	2.07724	78.30039	79.41448	79.42367
	S_1	2.07663	2.09191	2.11026	2.09997	2.08016	2.07351	77.59818	78.81319	80.41949
	T_1	2.10181	2.07952	2.06820	2.07414	2.10412	2.11929	77.46139	80.34703	78.54034
$[HL]^{3+}$	GS	2.06924	2.06896	2.07635	2.08182	2.08045	2.07729	78.48967	79.43887	79.44460
	S_1	2.09480	2.08100	2.07383	2.07902	2.09753	2.10880	77.75522	80.43150	78.84759
	T_1	2.10410	2.08557	2.06938	2.07416	2.10103	2.11692	77.51265	80.40983	78.60315

$[H_2L]^{4+}$	GS	2.06823	2.06777	2.07811	2.08123	2.08153	2.07779	78.52250	79.41610	79.43050
	S_1	2.06267	2.06185	2.09141	2.11688	2.11582	2.09041	79.48009	78.83540	78.84779
	T_1	2.05806	2.05811	2.09602	2.12498	2.12256	2.09440	79.47858	78.67088	78.73266
$[H_3L]^{5+}$	GS	2.06812	2.06424	2.08000	2.08185	2.08089	2.08010	78.67833	79.40819	79.41505
	S_1	2.07034	2.06649	2.08965	2.10928	2.10984	2.09342	79.49751	78.99651	78.90211
	T_1	2.06135	2.06419	2.09488	2.11838	2.11709	2.09532	79.48712	78.76394	78.81798
$[H_4L]^{6+}$	GS	2.06187	2.06359	2.08163	2.08310	2.08089	2.08110	78.66741	79.38489	79.42433
	S_1	2.07222	2.07031	2.09116	2.10734	2.10963	2.09179	79.22582	79.01285	78.94431
	T_1	2.06498	2.06581	2.09495	2.11640	2.11796	2.09436	79.25108	78.84471	78.79026
$[H_5L]^{7+}$	GS	2.06183	2.06356	2.08174	2.08241	2.08222	2.08225	78.65663	79.35268	79.40548
	S_1	2.07337	2.07149	2.09230	2.10638	2.10839	2.09345	79.12841	78.97017	78.99623
	T_1	2.06766	2.06591	2.09546	2.11531	2.11716	2.09730	79.12381	78.82230	78.81827

Table S5. Absolute, consecutive and relative (**to** the L^{2+} ligand) values of the vertical Ru(II) binding energies at the coordination centre, $\Delta E_{binding}$, of the studied species, in kcal/mol.

	L^{2+}	$[HL]^{3+}$	$[H_2L]^{4+}$	$[H_3L]^{5+}$	$[H_4L]^{6+}$	$[H_5L]^{7+}$	L'
absolute $\Delta E_{binding}$	-268.2	-267.2	-264.4	-258.5	-254.6	-254.0	-271.7
consecutive $\Delta E_{binding}$		1.0	2.8	5.9	3.9	0.6	
relative $\Delta E_{binding}$			3.8	9.7	13.6	14.2	-3.5

Table S5. Calculated contribution (in %) of the Ru *d*-orbitals (Ru), 1,10-phenantroline (Phen), and 2,2'-bipyridilophane (BPP) to the frontier orbitals (HOMO-2, HOMO-1, HOMO, LUMO, LUMO+1, LUMO+2) of L^{2+}, its protonated forms, and macrocycle-free derivative (L') in the singlet ground states.

	HOMO-2			HOMO-1			HOMO			LUMO			LUMO+1			LUMO+2		
	Ru	Phen	BPP	Ru	Phen	BPP	Ru	Phen	BPP	Ru	Phen	BPP	Ru	Phen	BPP	Ru	Phen	BPP
L'	75	13	12	73	24	3	86	10	4	43	55	2	50	41	9	0	93	7
L^{2+}	73	22	5	3	0	97	83	11	6	2	80	18	8	92	0	10	17	73
$[HL]^{3+}$	75	13	12	74	23	3	84	11	5	3	72	25	10	49	41	10	67	23
$[H_2L]^{4+}$	75	13	12	73	24	3	84	11	5	3	45	52	9	51	40	7	92	1
$[H_3L]^{5+}$	74	13	13	72	25	3	83	12	5	6	4	90	6	90	4	6	94	0
$[H_4L]^{6+}$	74	13	13	71	26	3	83	13	4	7	0	92	5	94	1	6	94	0
$[H_5L]^{7+}$	74	13	13	71	26	3	84	13	3	7	0	92	5	94	1	6	94	0

Table S6. Gibbs free energies computed in n-octanol and water SMD solvent, and resultant partition coefficients.

	$\Delta G°_o$ [au]	$\Delta G°_w$ [au]	$\Delta G°_{o/w}$ [au]	$\Delta G°_{o/w}$ [kcal/mol]	logP
L'	-1731.073869	-1731.067341	-0.006528	-4.1	3.01
L^{2+}	-2398.613068	-2398.608162	-0.004906	-3.1	2.26
$[HL]^{3+}$	-2399.057748	-2399.067134	0.009386	5.9	-4.33
$[H_2L]^{4+}$	-2399.485453	-2399.519489	0.034036	21.4	-15.74
$[H_3L]^{5+}$	-2399.879529	-2399.957244	0.077715	48.8	-35.87
$[H_4L]^{6+}$	-2400.264872	-2400.390613	0.125741	78.9	-58.04
$[H_5L]^{7+}$	-2400.625910	-2400.816664	0.190754	119.7	-88.04

Table S7. Main singlet vertical excitation energies (ΔE), absorption wavelengths (λ), oscillator strengths (*f*) and major transitions for the species studied in water, computed at the M06/6-31+G(d)/SDD level of theory.

Species	Excited State	ΔE (eV)	λ (nm)	f	Transitions	Theoretical Assignment
L'	S_1	2.50	495.49	0.001	H → L (30.9%)<br>H → L+1 (65.5%)	MLCT
	S_9	2.87	432.38	0.050	H-2 → L (5.3%)<br>H-2 → L+1 (32.0%)<br>H → L+3 (59.3%)	MLCT
	S_{12}	3.09	401.78	0.068	H-2 → L+3 (92.4%)<br>H-1 → L+4 (2.0%)<br>H → L+4 (2.5%)	MLCT + IL
L^{2+}	S_1	2.51	494.69	0.000	H → L+1 (94.9%)	MLCT
	S_9	2.79	443.93	0.160	H-3 → L+2, 50.8%<br>H → L+3, 18.9%<br>H-2 → L+1, 12.1%<br>H-3 → L, 8.6%<br>H-2 → L+2, 2.9%	MLCT
	S_{12}	3.04	408.45	0.067	H-3 → L+3, 90.0%<br>H → L+4, 2.8%	MLCT
$[HL]^{3+}$	S_1	2.52	492.25	0.000	H → L+2, 88.3%<br>H → L, 8.2%	MLCT
	S_9	2.85	434.35	0.131	H → L+3, 30.3%<br>H-2 → L+1, 29.5%<br>H-2 → L+2, 19.9%<br>H-2 → L, 8.2%<br>H-1 → L+2, 6.3%	MLCT
	S_{12}	3.05	406.72	0.066	H-2 → L+3, 90.9%<br>H → L+4, 2.9%	MLCT + IL
$[H_2L]^{4+}$	S_1	2.50	496.69	0.001	H → L+1, 55.9%<br>H → L, 40.1%	MLCT
	S_7	2.84	437.07	0.125	H-1 → L+2, 42.9%<br>H-2 → L+1, 37.7%<br>H-2 → L, 11.6%<br>H → L+3, 2.7%	MLCT
	S_8	2.85	434.57	0.113	H-2 → L+2 48.8%<br>H-1 → L+1, 35.8%<br>H-1 → L, 5.6%<br>H → L+4, 4.5%	MLCT + IL
	S_{12}	3.09	401.65	0.065	H-2 → L+3, 92.4%<br>H → L+4, 2.7%	MLCT + IL
$[H_3L]^{5+}$	S_1	2.33	532.86	0.000	H → L, 91.0%<br>H → L+1, 6.2%	MLCT
	S_5	2.72	456.24	0.160	H-2 → L, 88.0%<br>H → L+2, 3.9%<br>H-1 → L+2, 2.5%	MLCT
	S_8	2.92	424.18	0.108	H-2 → L+2, 53.6%<br>H-1 → L+1 , 31.7%<br>H-1 → L+2, 5.2%<br>H → L+4, 3.9%<br>H-2 → L+1, 2.2%	MLCT+IL
$[H_4L]^{6+}$	S_1	2.219	558.7	0.002	H → L 94.8%<br>H → L+1, 2.5%	MLCT
	S_3	2.64	469.79	0.142	H-2 → L, 82.0%<br>H → L+2, 8.3%<br>H-1 → L+2, 3.1%	MLCT
	S_8	2.98	415.54	0.093	H-2 → L+2, 61.6%<br>H-1 → L+1, 25.7%<br>H → L+3, 4.9%	MLCT + IL
$[H_5L]^{7+}$	S_1	2.193	565.3	0.003	H → L, 95.4%<br>H → L+1, 2.0%	MLCT
	S_3	2.6226	472.75	0.150	H-2 → L, 86.3%<br>H → L+2, 4.7%<br>H-1 → L+2, 2.8%	MLCT
	S_8	2.99	414.19	0.089	H-2 → L+2, 64.9%<br>H-1 → L+1, 23.8%<br>H → L+3, 3.9%	MLCT + IL

Table S8. Comparison between the linear response, the corrected linear response, and the external iteration derived absorption excitation wavelengths of the first band (experimental 450nm).

	L^{2+}	$[HL]^{3+}$	$[H_2L]^{4+}$	$[H_3L]^{5+}$	$[H_4L]^{6+}$	$[H_5L]^{7+}$
λ_{LR}	433.92	434.35	437.07	456.24	469.79	472.75
λ_{CLR}	433.72	433.51	435.78	457.91	471.74	475.34
λ_{ExI}	502.64	462.29	423.16	403.84	370.95	353.75

Table S9. Transitions corresponding to the generation of accessible triplet states (T_1) for studied species in water, computed at the M06/6-31+G(d)/SDD level of theory.

Species	T_m	Transitions	Theoretical assignment	Species	T_m	Transitions	Theoretical assignment
L'	T_1	H-4 → L+3, 3.3%<br>H-3 → L+4, 4.6%<br>H-2 → L+1, 2.5%<br>H-1 → L+2, 5.2%<br>H-1 → L+3, 7.5%<br>H → L+2, 64.1%	$d_{z^2}\rightarrow\pi^*_{Phen}\pi^*_{Phen}\pi^*_{BPP}$				
	T_2	H-1 → L+4, 2.6%<br>H → L+1, 77.9%	$d_{z^2}\rightarrow\pi^*_{Phen}\pi^*_{Phen}\pi^*_{BPP}$				
	T_3	H-4 → L+4, 2.0%<br>H-3→ L+3, 3.1%<br>H-2 → L+2, 2.4%<br>H-2 → L+3, 2.5%<br>H-1 → L+4, 5.8%<br>H → L, 72.1%	$d_{z^2}\rightarrow\pi^*_{Phen}\pi^*_{Phen}\pi^*_{BPP}$				
	T_4	H-2→ L+1, 38.2%<br>H-1 → L+2, 50.7%<br>H → L+3, 3.9%	$d_{z^2}\rightarrow\pi^*_{Phen}\pi^*_{Phen}$				
	T_5	H-2→ L+2, 21.4%<br>H-1 → L, 25.4%<br>H-1 → L+1, 41.6%<br>H→ L+4, 3.0%	$d_{z^2}\rightarrow\pi^*_{Phen}\pi^*_{Phen}\pi^*_{BPP}$				
L^{2+}	T_1	H → L+1, 65.0%<br>H-2 → L+3, 6.3%<br>H-2 → L+1, 4.2%<br>H-9 → L+4, 4.0%<br>H-8 → H+3, 2.9%<br>H-2 → L, 2.3%	$d_{z^2}\rightarrow\pi^*_{Phen}$	$[H_3L]^{5+}$	T_1	H → L, 78.2%<br>H → L+1, 7.7%<br>H-2 → L, 3.5%	$d_{z^2}\rightarrow\pi^*_{Phen}$
	T_2	H → L+2 (34.4%)	$d_{z^2}\rightarrow\pi^*_{BPP}$		T_2	H-2 → L (73.8%)	$d_{t^2g}\rightarrow\pi^*_{Phen}$
	T_3	H-2 → L+1 (34.4%)	$d_{t^2g}\rightarrow\pi^*_{Phen}\pi^*_{Phen}$				
	T_4	H → L+2 (46.7%)	$d_{z^2}\rightarrow\pi^*_{BPP}$				
	T_5	H-2 → L (40.9%)	$d_{t^2g}\rightarrow\pi^*_{Phen}\pi^*_{Phen}\pi^*_{BPP}$				
$[HL]^{3+}$	T_1	H → L+2, 40.3%<br>H → L+1, 24.5%<br>H-1 → L+3, 7.1%<br>H-7 → L+4, 4.3%<br>H-1 → L+2, 3.3%<br>H-8 → L+3, 3.2%<br>H-2 → L, 2.1%	$d_{z^2}\rightarrow\pi^*_{Phen}\pi^*_{BPP}$	$[H_4L]^{6+}$	T_1	H → L, 86.0%<br>H-1 → L, 3.3%<br>H → L+1, 3.2%	$d_{z^2}\rightarrow\pi^*_{Phen}$
	T_2	H → L+1 (31.0%)	$d_{z^2}\rightarrow\pi^*_{Phen}\pi^*_{BPP}$		T_2	H-2 → L (86.0%)	$d_{t^2g}\rightarrow\pi^*_{Phen}$
	T_3	H → L (35.2%)	$d_{z^2}\rightarrow\pi^*_{Phen}\pi^*_{Phen}\pi^*_{BPP}$				
	T_4	H-1 → L+2 (27.8%)	$d_{t^2g}\rightarrow\pi^*_{Phen}\pi^*_{BPP}$				
	T_5	H-1 → L (37.1%)	$d_{t^2g}\rightarrow\pi^*_{Phen}\pi^*_{Phen}\pi^*_{BPP}$				
	T_6	H-2 → L+1 (41.1%)	$d_{t^2g}\rightarrow\pi^*_{Phen}\pi^*_{BPP}$				

Complex	State	Transition	Character
$[H_2L]^{4+}$	T_1	H → L+1, 72.2%<br>H → L, 6.9%	$d_{z^2}\rightarrow\pi^*_{Phen}\pi^*_{Phen}\pi^*_{BPP}$
	T_2	H → L+2 (61.2%)	$d_{z^2}\rightarrow\pi^*_{Phen}\pi^*_{Phen}$
	T_3	H → L (64.1%)	$d_{z^2}\rightarrow\pi^*_{Phen}\pi^*_{Phen}\pi^*_{BPP}$
	T_4	H-1 → L+2 (43.1%)	$d_{t^2g}\rightarrow\pi^*_{Phen}\pi^*_{Phen}$
	T_5	H-2 → L (37.0%)	$d_{t^2g}\rightarrow\pi^*_{Phen}\pi^*_{Phen}\pi^*_{BPP}$
$[H_5L]^{7+}$	T_1	H → L, 87.6%<br>H-1 → L, 2.7%<br>H → L+1, 2.7%	$d_{z^2}\rightarrow\pi^*_{Phen}$
	T_2	H-2 → L (88.0%)	$d_{t^2g}\rightarrow\pi^*_{Phen}$

Table S10. Main bond lengths (in Å) and angles (in degrees) for the protonated ground states of copper complexes computed at the PBE1PBE/6-31G(d) level of theory in water.

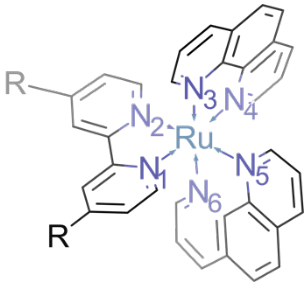

	Ru-N_1	Ru-N_2	Ru-N_3	Ru-N_4	Ru-N_5	Ru-N_6	N_1-Ru-N_2	N_3-Ru-N_4	N_5-Ru-N_6
$[CuL]^{4+}$	2.07650	2.07516	2.07793	2.07722	2.07884	2.07891	78.88758	79.47498	79.46652
$[HCuL]^{5+}$	2.07547	2.07912	2.08578	2.08558	2.08281	2.08575	78.46493	79.32406	79.31502
$[H_2CuL]^{6+}$	2.09056	2.09618	2.08357	2.07692	2.07452	2.08575	78.17389	79.62366	79.39406
$[H_3CuL]^{7+}$	2.07895	2.08410	2.08527	2.08187	2.08196	2.08139	78.37960	79.37722	79.43753

FIGURES

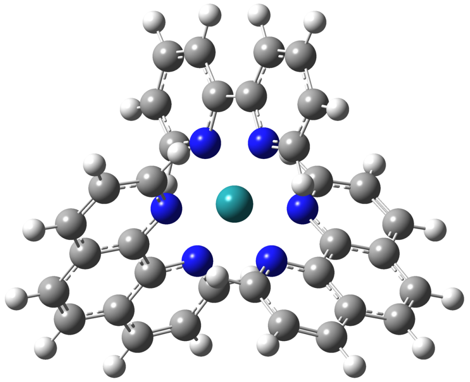

Figure S1. Optimised structure of the L' compound.

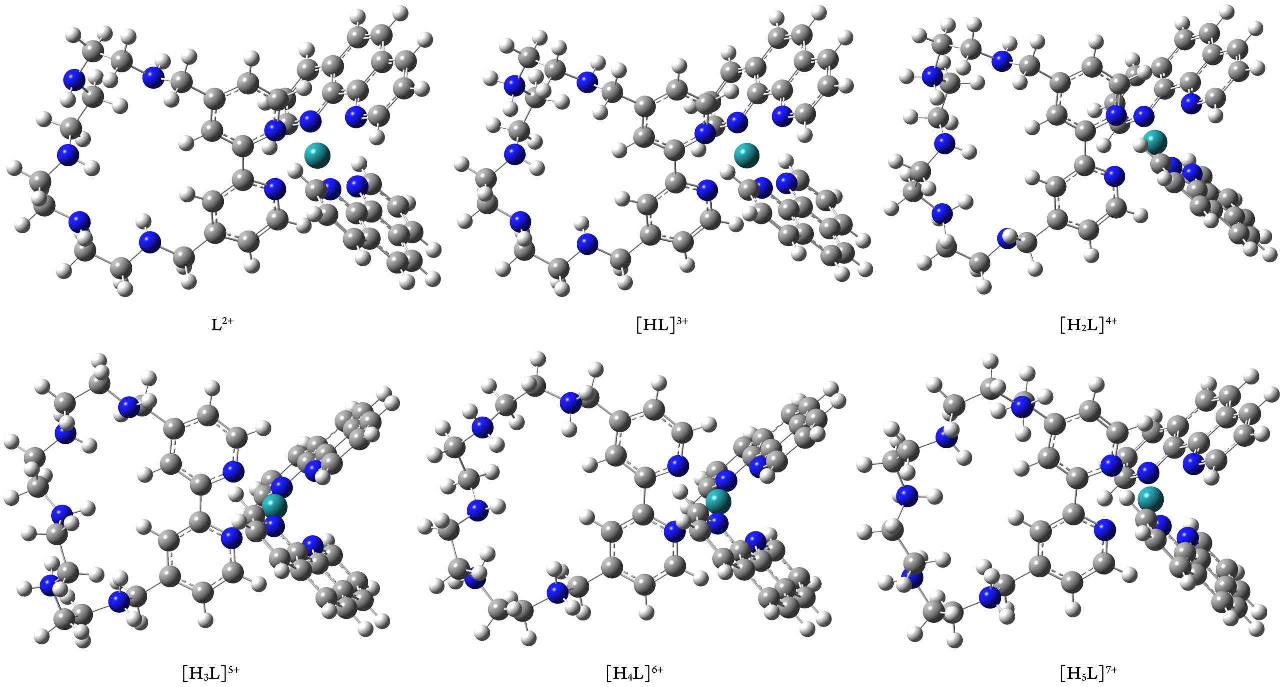

Figure S2. Conformation of the L^{2+} ground state and its protonated forms.

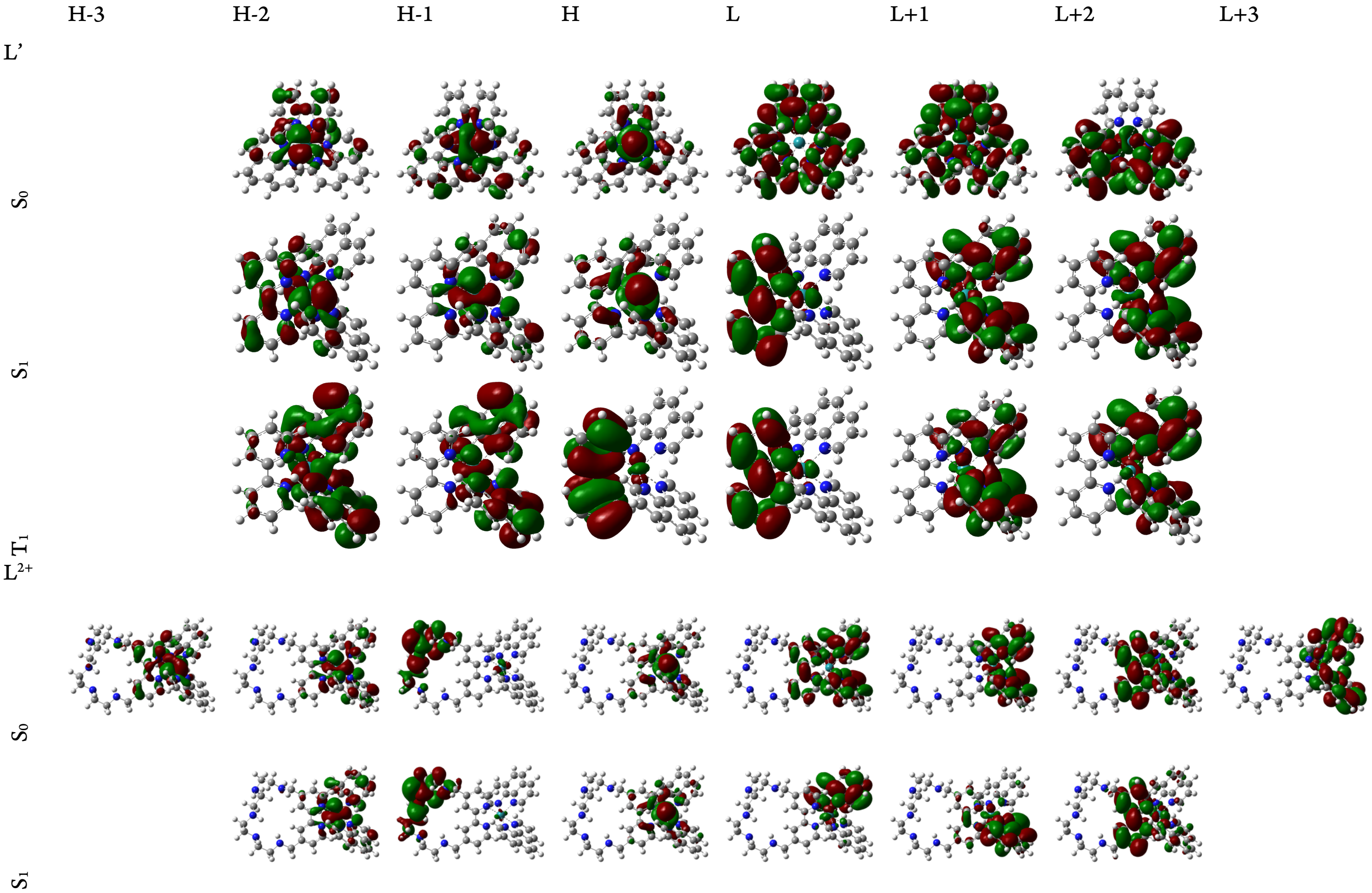

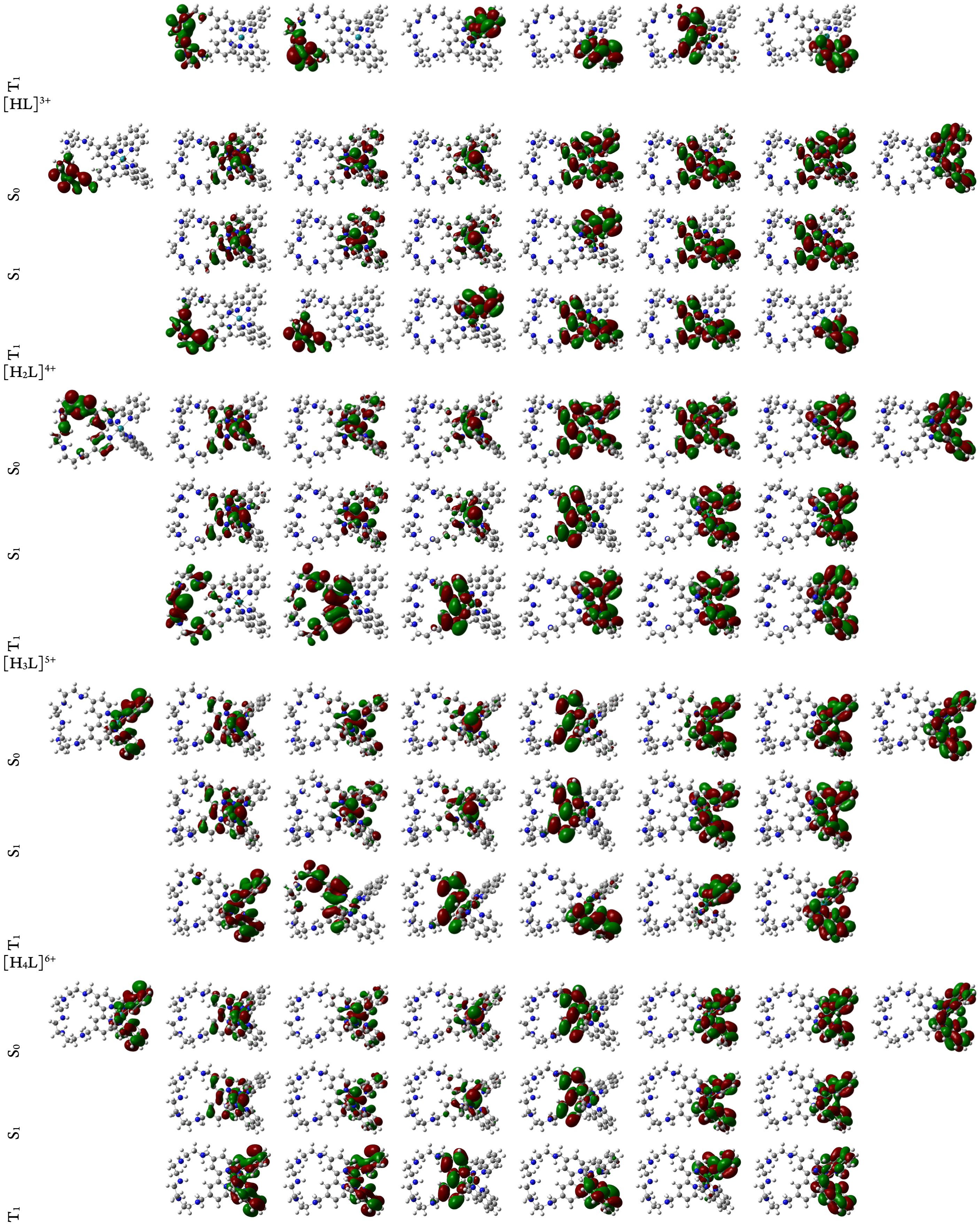

T_1
$[HL]^{3+}$
S_0
S_1
T_1
$[H_2L]^{4+}$
S_0
S_1
T_1
$[H_3L]^{5+}$
S_0
S_1
T_1
$[H_4L]^{6+}$
S_0
S_1
T_1

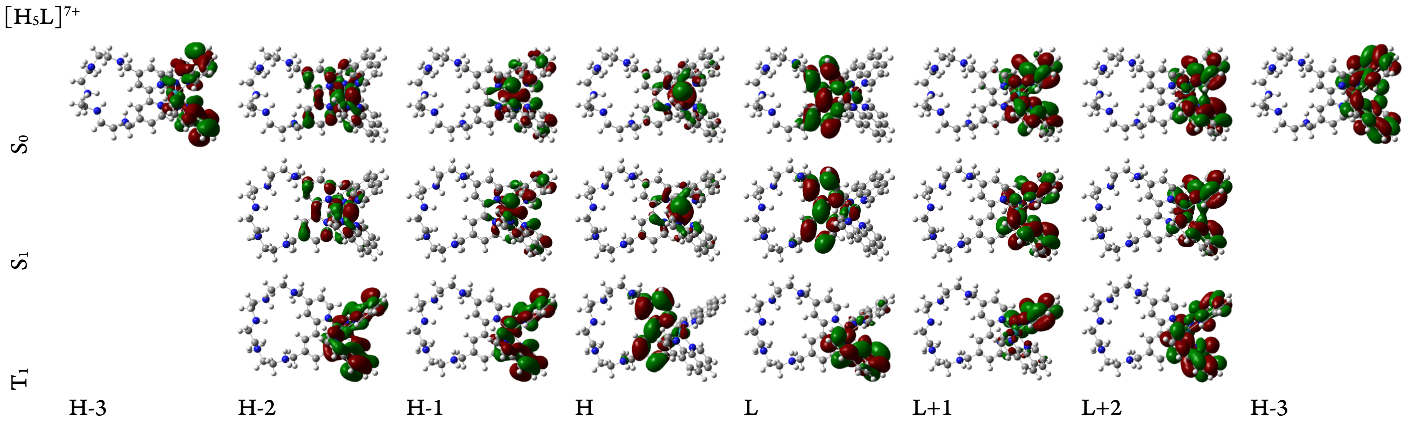

Figure S3. MOs of all species in their ground, low-lying singlet and low-lying triplet states.

		α-spin	β-spin
$[CuL]^{4+}$	LUMO	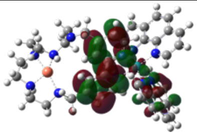	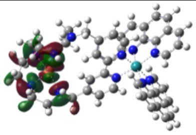
		-2.55eV	-3.65eV
	SOMO	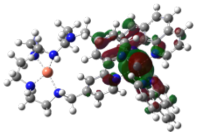	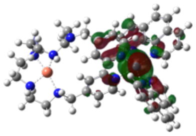
		-6.32eV	-6.33eV
	$\Delta E_{S\text{-}L}$	3.77eV	2.68eV
$[HCuL]^{5+}$	LUMO	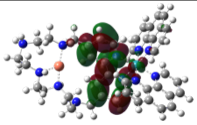	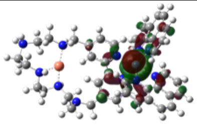
		-3.08eV	-4.95eV
	SOMO	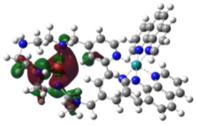	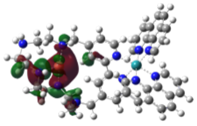
		-6.35eV	-6.35eV
	$\Delta E_{S\text{-}L}$	3.27eV	1.40eV
$[H_2CuL]^{6+}$	LUMO	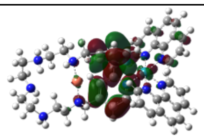	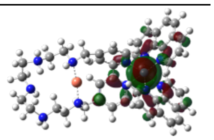
		-3.33eV	-5.09eV
	SOMO	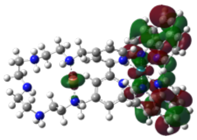	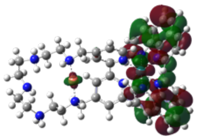
		-7.72eV	-7.67eV
	$\Delta E_{S\text{-}L}$	4.39eV	2.58eV

$[H_3CuL]^{7+}$	LUMO	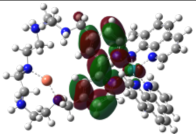	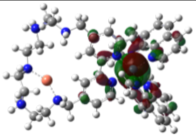
		-3.44eV	-5.11eV
	SOMO	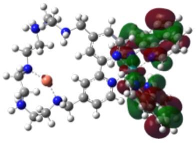	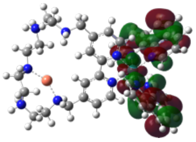
		-7.75eV	-7.70eV
	ΔE_{S-L}	4.31eV	2.59eV

Figure S4. Graphical representation of the Cu^{2+}-complex singlet occupied and lowest unoccupied molecular orbitals categorised according to the protonation state
